# Contribution of underlying processes to improved visuospatial working memory associated with physical activity

**DOI:** 10.7717/peerj.3430

**Published:** 2017-06-06

**Authors:** Qingchun Ji, Yingying Wang, Wei Guo, Chenglin Zhou

**Affiliations:** Department of Sport Psychology, School of Sport Science, Shanghai University of Sport, Shanghai, Asia, China

**Keywords:** Physical activity, Visuospatial working memory, Storage, Manipulation, Sex difference

## Abstract

**Background:**

Working memory is critical for various cognitive processes and can be separated into two stages: short-term memory storage and manipulation processing. Although previous studies have demonstrated that increased physical activity (PA) improves working memory and that males outperform females on visuospatial working memory tasks, few studies have determined the contribution of the two underlying stages to the visuospatial working memory improvement associated with PA. Thus, the aims of the present study were to verify the relationship between physical activity and visuospatial working memory, determine whether one or both stages were affected by PA, and investigate any sex differences.

**Methods:**

A total of 56 undergraduate students were recruited for this study. Their scores on the International Physical Activity Questionnaire (IPAQ) were used to separate them into either a lower PA (*n* = 26; IPAQ score ≤3,000 metabolic equivalent [MET]-min/week) or higher PA (*n* = 30; IPAQ score >3,000 MET-min/week) group. Participants were required to complete three tasks: a visuospatial working memory task, a task that examines the short-term memory storage stage, and a mental rotation task that examines the active manipulation stage.

**Results:**

Participants in the higher PA group maintained similar accuracy but displayed significantly faster reaction times (RT) than those in the lower PA group on the visuospatial working memory and manipulation tasks. By contrast, no difference was observed between groups on the short-term memory storage task. In addition, no effects of sex were detected.

**Discussion:**

Our results confirm that PA was positively to visuospatial working memory and that this positive relationship was associated with more rapid cognitive processing during the manipulation stage, with little or no relationship between PA and the memory storage stage of visuospatial working memory.

## Introduction

Physical activity (PA) is associated with improved cognitive functions (Hötting & Röder, 2013; Sibley & Etnier, 2003), such as working memory (Kamijo et al., 2011). Working memory, which refers to the ability to temporarily store and manipulate information (Baddeley et al., 1999; Pontifex et al., 2009) that underpins many cognitive processes (Paulus & Yu, 2012), consists of a central executive system and two independent slave subsystems: the phonological loop and the visuospatial sketchpad (Baddeley, 1998). According to the “continuum” model proposed by Vecchi & Cornoldi (1999), visuospatial working memory is divided into the passive storage and active manipulation stages based on the degree of active information processing. Passive storage refers to the short-term maintenance processing of visuospatial information, and active manipulation refers to the mental manipulation processing of the stored information. The present study aimed to determine whether a relationship exists between PA and visuospatial working memory and, if so, which of these two memory processing stages underlie that relationship.

Many studies have demonstrated that participation in PA leads to better verbal working memory. For example, following a 9-month afterschool PA intervention, preadolescent children exhibit improved response accuracy in letter recognition, with beneficial effects greater for more difficult task conditions (Kamijo et al., 2011). However, the working memory tasks used in such studies have mainly tested storage processing with a low load of active manipulation processing (Schooler et al., 2008). By contrast, the present study used two distinct tasks, with one tapping into the passive storage stage of memory processing and the other into the active manipulation stage (Suchan et al., 2002). We used the same visuospatial working memory task, short-term storage task, and mental rotation task that we had used in our previous study to measure general visuospatial working memory as well as the passive storage and the active manipulation stages, respectively (Guo et al., 2016). On the basis of the results of previous studies suggesting a relationship between PA and short-term memory (Cooper et al., 2012; Kamijo et al., 2011; Roig et al., 2013), we hypothesized that participants who were classified as more physically active would exhibit superior general visuospatial working memory task performance and that PA would thus be significantly associated with the performance of at least the storage stage.

Sex differences in visuospatial processing have been discussed for a long time. Neuroimaging studies have found that sex differences in cerebral activity distribution are more prominent while participants are performing a mental rotation task rather than a verbal-related task (Gizewski et al., 2006; Hugdahl, Thomsen & Ersland, 2006). Generally, males outperform females on this kind of visuospatial task (Lauer et al., 2015; Voyer, Voyer & Bryden, 1995). Additionally, a recent meta-analysis demonstrated a male advantage in visuospatial working memory (Voyer, Voyer & Saint-Aubin, 2016). Some studies have proposed that the modulatory effects of sex steroids may be responsible for the observed sex differences in visuospatial performance (Mäntylä, 2013; Courvoisier et al., 2013; Thilers, MacDonald & Herlitz, 2006). Therefore, the variable of sex was also considered in the present study, especially the potential effects of sex on the manipulation stage. As a first step for future longitudinal studies, the present study used a cross-sectional design to preliminarily determine whether a relationship existed between PA and the two stages of visuospatial working memory in males and females. Participants were divided into four groups based on their level of PA and their sex and then completed the three tasks. We expected that the group comprising males with the higher PA would show better visuospatial working memory in general and would be better at the task tapping into the manipulation stage of visuospatial memory processing than the three other groups.

## Methods

### Ethical approval

The study was carried out ethically and approved by the Ethical Committee of Shanghai University of Sport (No. 2015003).

### Participants

A total of 56 college students (sex, 36% females; age, 21.68 ±1.63 years; 18.5  ≤ BMI ≤24.99 kg/m^2^) recruited through advertisements voluntarily participated in the present study. All participants gave their informed consent. The study protocol was approved by the ethics committee of Shanghai University of Sport, and the study was conducted according to the Declaration of Helsinki’s guidelines. No participants had consumed alcohol or drugs, including prescription medication, known to affect cognition in the preceding year.

**Table 1:** Participant characteristics presented by physical activity group.

Variable	LG	HG	*t* or *χ*^2^	*p*
*n*	26	30		
Age (years)	21.50 ± 1.39	21.57 ± 1.41	0.18	0.860
Sex (% female)	38%	33%	0.16	0.690
BMI (kg/m^2^)	20.74 ± 1.61	21.12 ± 1.53	−0.91	0.368
IPAQ (METs)^**^	1529.50 ± 806.46	6895.33 ± 2932.59	−9.61	<0.001

**Notes.**

Values indicate mean ± standard deviation.

BMI body mass index IPAQ International Physical Activity Questionnaire HG higher activity group LG lower activity group

** Indicates significance difference between the two groups, *p* < 0.01.

Based on their International Physical Activity Questionnaire (IPAQ, Taiwan Version; Liou et al., 2008) scores, participants were separated into higher activity groups (HGs) and lower-activity groups (LGs). Participants in the HG scored more than 3,000 metabolic equivalent (MET)-min/week, whereas those in the LG scored equal to or less than 3,000 MET-min/week (IPAQ Research Committee, 2005). Therefore, twenty-six participants were included in the LG and thirty in the HG. All 56 participants completed the general visuospatial working memory, short-term storage, and mental manipulation tasks. The demographic and activity characteristics for the two groups are shown in Table 1.

### Tasks

**Figure 1:**
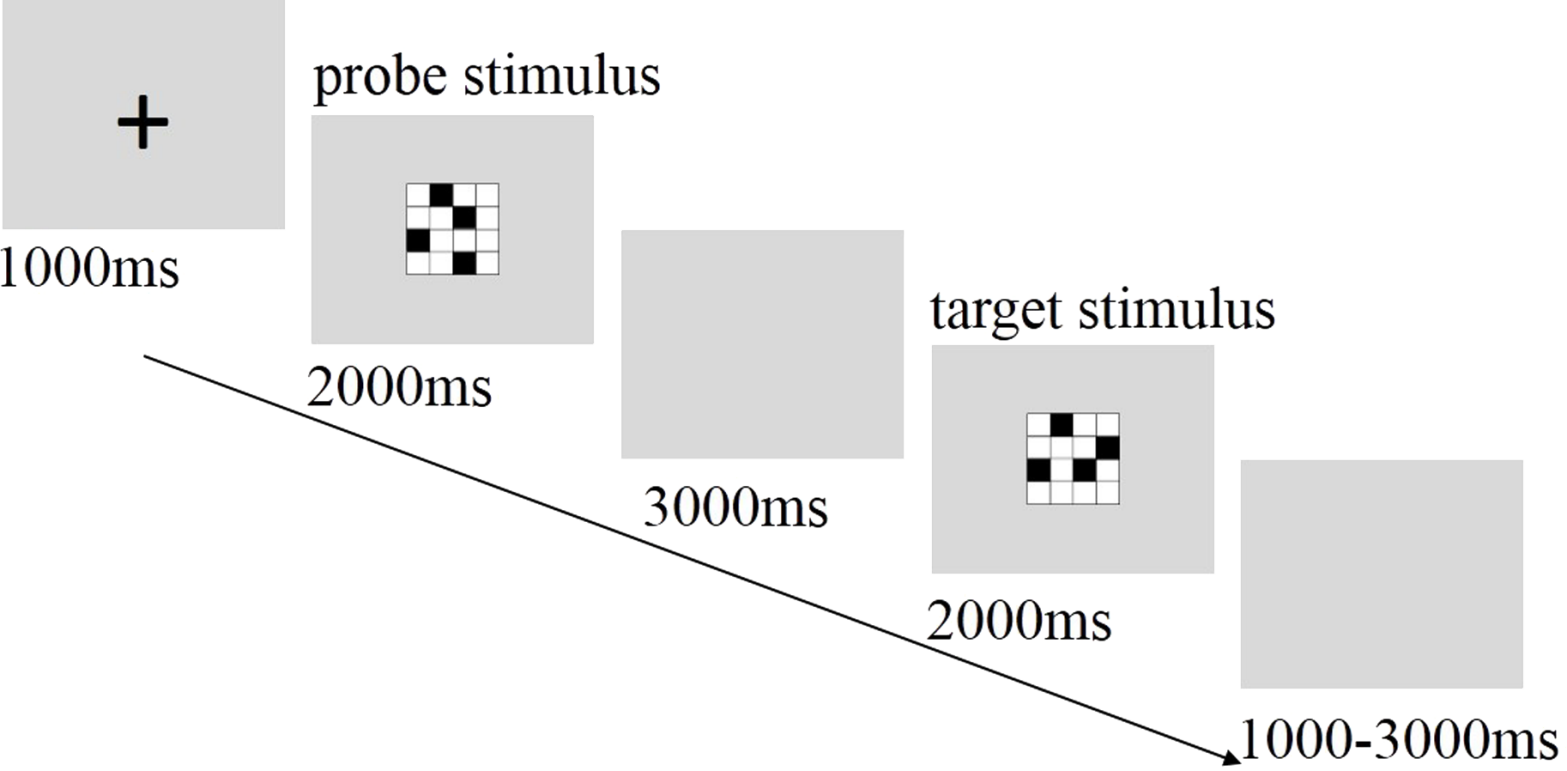
Sequence of events within a single trial of the visuospatial working memory task.

The three tasks were programmed using Matlab R2011a, Psychtoolbox 3.0 (MathWorks, Natick, USA), and each participant completed all trials. All stimuli were matrices consisting of 4 × 4 squares (Suchan et al., 2006), with each matrix containing 4 black squares and 12 white squares (see Fig. 1). The position of the 4 black squares within the matrix was altered for each different stimulus. Stimuli were presented in the center of a 17-inch NESO computer monitor with a vertical visual angle of 2.8°. The order for each task was counterbalanced across all participants.

#### Visuospatial working memory task (VST)

The VST was used to test general visuospatial working memory (Suchan et al., 2006). Participants were asked to recognize whether the presented matrix (target stimulus) was an exact 90° rotation (to left or right) of the last presented matrix (probe stimulus). To indicate 90° rotation, participants pressed the “1” key on a numeric keyboard with their left index finger, and to indicate no such rotation, they pressed “3” with their right index finger. The task included 40 randomized trials in which matched matrices were presented with 50% probability. Each trial started with a fixation point presented for 1,000 ms on the center of the screen. The probe stimulus was then presented for 2,000 ms, followed by a gray blank. Subsequently, participants were instructed to respond to the target stimulus within 3,000 ms. The experimental flow is shown in Fig. 1.

#### Short-term storage task (SST)

**Figure 2:**
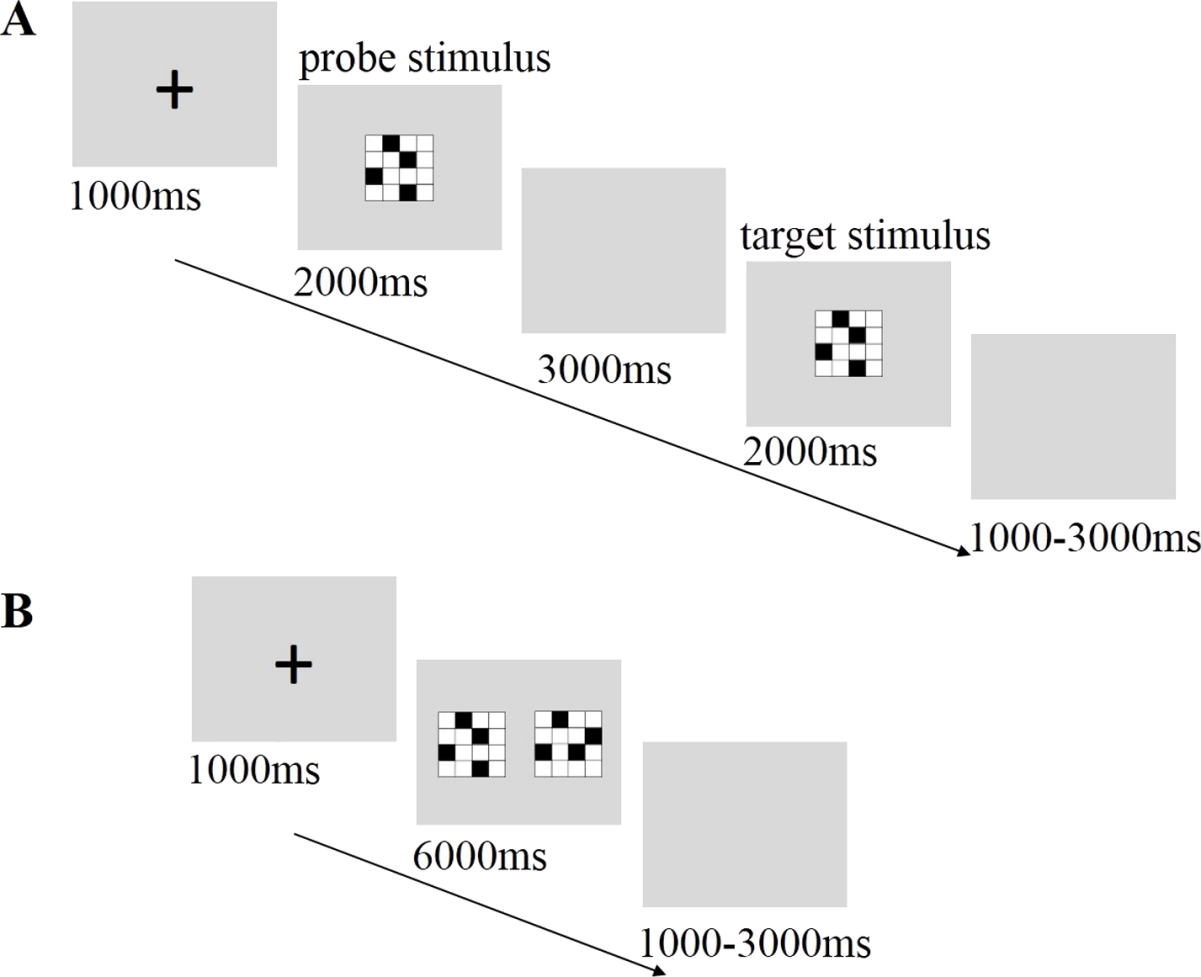
Sequence of events within a single trial of the short-term storage task (A) and mental rotation task (B).

The SST was used to access the storage processing stage of visuospatial working memory (Suchan et al., 2006). The task required participants to respond as quickly as possible to target stimuli. During the blank stimulus that was presented between the probe and target stimuli presentations, the participants had to maintain a mental image of the probe stimulus and compare it with the target stimulus. When the probe and target stimuli were identical, participants pressed the “1” key with their left index finger. When the position of the four black squares within the matrix differed from that of the probe stimulus, participants pressed the “3” key with their right index finger. The presentation time and order of the stimuli were the same as those used in the VST (see Fig. 2). The task consisted of 40 trials in which the matched probability was 50%.

#### Mental rotation task (MRT)

The MRT is regarded as a promising method for testing the manipulation processing of visuospatial working memory (Seurinck et al., 2004). The participants were required to compare two simultaneously presented matrices and to mentally rotate the matrices. They determined whether or not the matrix on the right was an exact 90° rotation of the matrix on the left. They then pressed the “1” or “3” key with their left or right index finger, respectively, to indicate if the matrix was or was not a match, respectively. After a fixation point was presented for 1,000 ms, two matrices were displayed at the center of the screen for 6,000 ms, and participants were required to respond within 3,000 ms (see Fig. 2). All 40 trials were presented with equal probability of being matched or unmatched.

### Procedure

The experiment employed a within-subjects design. Each participant was tested in a quiet room and completed all three tasks in a counterbalanced order. During the first visit to the laboratory, participants completed the informed consent, demographics questionnaire, and IPAQ to determine their body mass index (BMI) and level of PA. On the second visit, participants were given instructions and the allowed to practice each of the three tasks. After the accuracy of the practice test for each task was greater than 60%, the participants started the tasks for data collection. Response accuracy and reaction time (RT) were collected.

### Statistical analysis

First, a *t*-test or χ^2^-test was used to access the differences across groups in age, percentage of females, BMI, and PA level. Statistical analyses were then conducted using Matlab for each task, which included removing data that were beyond three standard deviations and outputting averaged data for each participant. A repeated-measures analysis of variance (ANOVA) was used to verify the different cognitive demands of the three tasks (VST, SST, and MRT) using SPSS v. 20 software (SPSS, Chicago, IL, USA). In order to analyze the differences in performance on the general visuospatial working memory, short-term storage, and mental rotation tasks across groups and sex, the RT and response accuracy were separately analyzed using a 2 (Group: LG, HG) ×2 (Sex: male, female) two-way ANOVA for each task. Regarding the RT, the results of the homogeneity of variance tests showed that the variance of the dependent variable was unequal across the four groups for both the VST (*F*_(3,52)_ = 3.38, *p* = 0.025) and the MRT (*F*_(3,52)_ = 3.30, *p* = 0.027) but was equal for the SST (*F*_(3,52)_ = 1.44, *p* = 0.241). Therefore, the Mann–Whitney *U* test was performed for the analysis of RT between groups for the VST and MRT. Regarding response accuracy, the variance of the dependent variable was equal across the four groups in all three tasks. Additionally, bivariate correlation was used to analyze the relationship between the levels of PA (scores of IPAQ) and the performance of each task. The significance level of all statistical analyses is *p* = 0.05.

## Results

### Comparison of demographic data and PA levels between LG and HG

The levels of PA significantly differed between groups, *t*_(54)_ =  − 9.61, *p* < 0.001, with HG exhibiting a higher level of PA relative to LG. However, no significant differences were observed between groups for age, sex, or BMI (Table 1).

**Figure 3:**
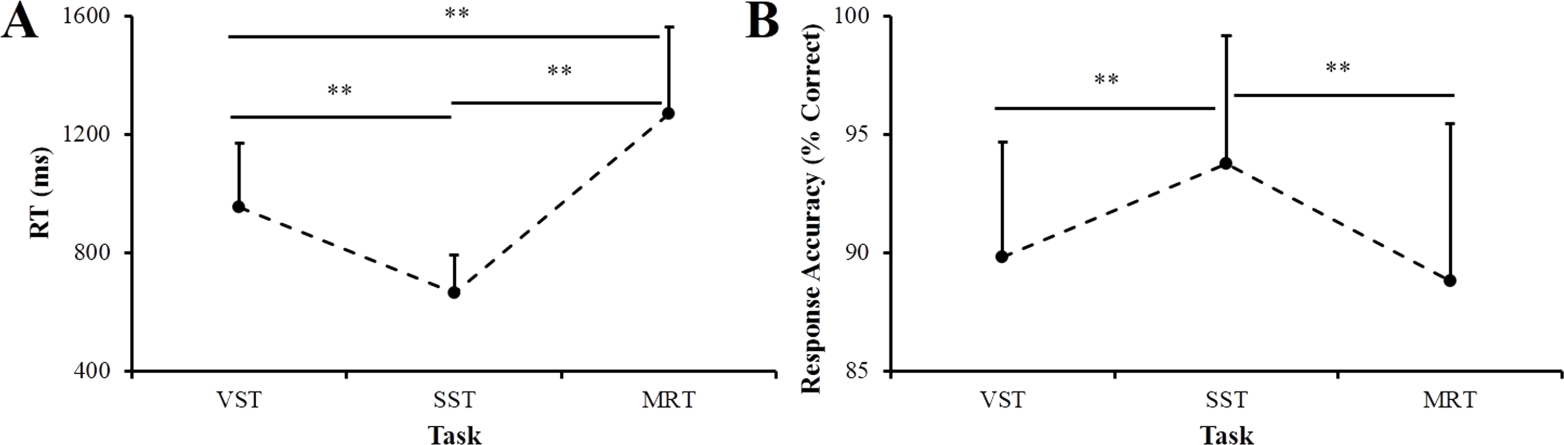
Reaction time (RT) (A) and response accuracy (B) of participants on three tasks. VST, visuospatial working memory task; SST, short-term memory storage task; MRT, mental rotation task. ^∗∗^*p* < 0.01 between the indicated tasks.

### The three tasks require different cognitive demands

A one-way (task: VST, SST, and MRT) within-subject design ANOVA examining RT revealed a significant main effect of task (*F*_(2,110)_ = 230.93, *p* < 0.001, η^2^ = 0.81). The results of post hoc tests found that the responses of the participants were slowest on the MRT (1268.9 ± 292.2 ms), faster on the VST (952.1 ± 218.9 ms), and fastest on the SST (665.0 ± 127.3 ms). Our analyses also revealed a significant effect of task on response accuracy (*F*(2,110) = 14.14, *p* < 0.001, η^2^ = 0.21). Post hoc test results indicated that the accuracy on the SST (93.8% ± 5.4%) was significantly greater than that on the other two tasks, which did not differ (VST: 89.8% ± 4.9%; MRT: 88.8% ± 6.7%) (Fig. 3).

Using regression analysis, we found that the RTs on the SST and on the MRT accurately predicted the RT on the VST (*F*_(2,53)_ = 32.65, *p* < 0.001). This result supports our finding that visuospatial working memory processing can be divided into storage and manipulation stages.

### PA is associated with general visuospatial working memory (Table 2)

For participant performance on the VST, the results of the Mann–Whitney *U* test showed that the HG exhibited a shorter RT than the LG (*Z* =  − 2.10, *p* = 0.035). No significant difference was observed in RT between males and females (*Z* =  − 1.71, *p* = 0.087). Two-way ANOVA—2 (Group: LG, HG) × 2 (Sex: male, female)—results for response accuracy showed no main effects of group or sex and no significant interactions.

**Table 2:** Reaction time and response accuracy by group of the participants during their performance on three tasks

Task	Measure	HG	LG	*p*
VST	RT (ms)^*^	885.00 ± 127.72	1029.47 ± 273.57	0.017
	Accuracy (%)	90.17 ± 5.04	89.42 ± 4.71	0.727
SST	RT (ms)	634.03 ± 112.94	700.71 ± 135.63	0.170
	Accuracy (%)	94.00 ± 6.45	93.46 ± 4.07	0.750
MRT	RT (ms)^*^	1166.67 ± 200.42	1386.85 ± 337.96	0.017
	Accuracy (%)	89.08 ± 6.11	88.46 ± 7.35	0.641

**Notes.**

Values are mean ± standard deviation.

HG higher activity group LG lower activity group RT reaction time VST visuospatial working memory task SST short-term storage task MRT mental rotation task

* Indicates significance difference between the two groups, *p* < 0.05.

For participant performance on the SST, two-way ANOVA—2 (Group: LG, HG) × 2 (Sex: male, female)—results showed no main effects of group or sex and no significant interactions for either RT or response accuracy.

For participant performance on the MRT, the results of the Mann–Whitney *U* test showed that the HG exhibited a shorter RT than the LG (*Z* =  − 2.43, *p* = 0.015). No significant difference was observed in RT between males and females (*Z* =  − 1.16, *p* = 0.245). The two-way ANOVA—2 (Group: LG, HG) ×2 (Sex: male, female)—results also found no significant main effects or interactions for response accuracy.

### PA is correlated with performance on the mental rotation task

**Figure 4:**
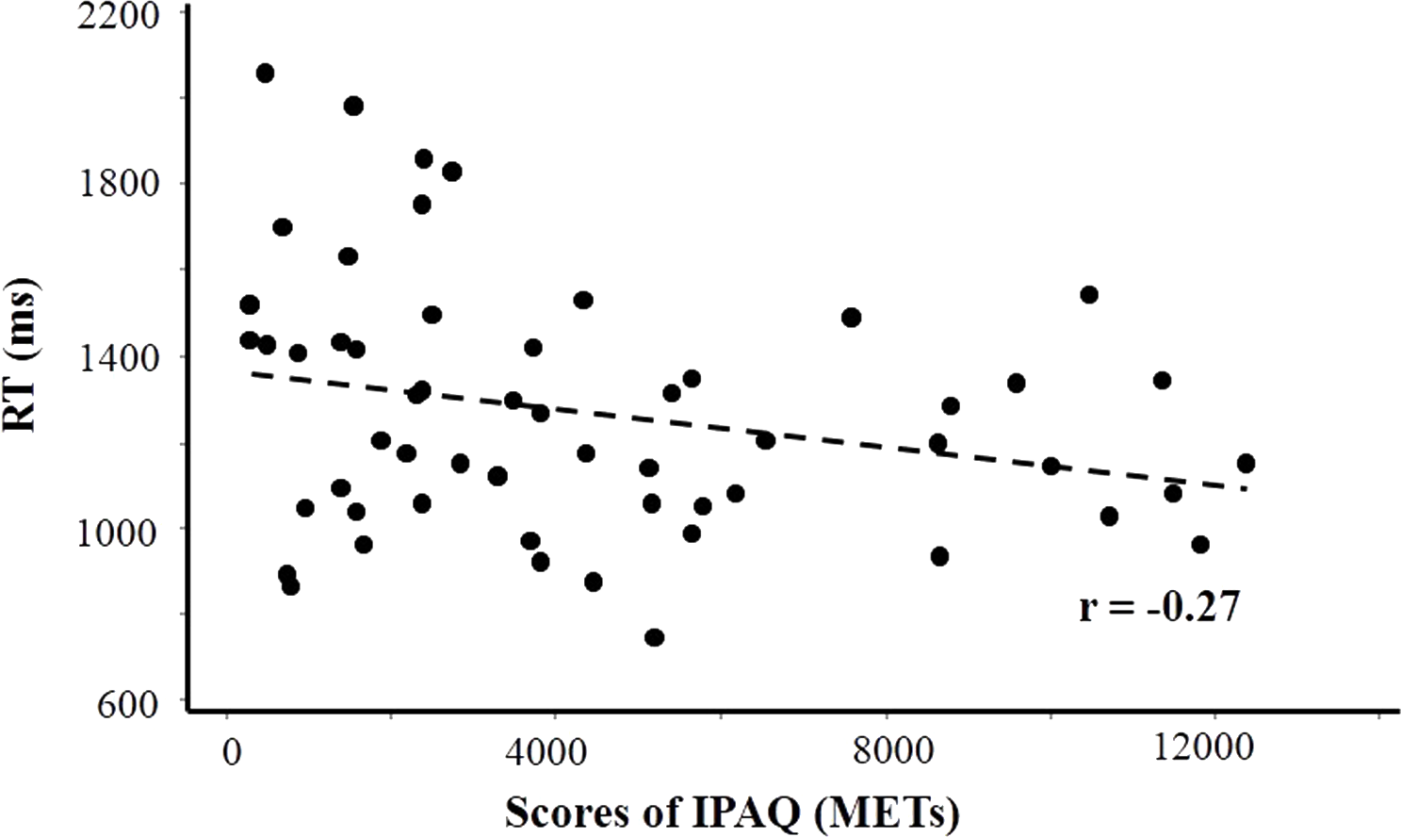
Scatter plot depicting a significant relationship between International Physical Activity Questionnaire (IPAQ) scores and reaction times (RT) for all participants performing the mental rotation task.

There was a significant correlation between the IPAQ scores and RT on the MRT as determined using a bivariate correlation analysis (*r* =  − 0.27, *p* = 0.044). We found that participants with higher PA responded faster during mental manipulation processing (Fig. 4). Although we examined potential correlations between IPAQ scores and the performance on each task, no other correlations were significant.

## Discussion

To determine whether a relationship existed between PA and visuospatial working memory and the potential contribution of the two stages underlying the processing of this type of memory, we investigated the differences between groups of healthy participants with high (HG) and low (LG) PA—grouped on the basis of their self-reported IPAQ scores—on their performance of three tasks. Consistent with the results of most previous studies, participants with higher PA demonstrated faster general visuospatial working memory than those with lower PA. When we further examined the contribution of the two stages underlying visuospatial working memory processing by analyzing the performance of participants on two tasks that distinguish these two stages, we found that the significant difference in visuospatial working memory between participants with different PA levels was associated with the mental manipulation stage rather than the memory storage processing stage. A significant correlation was detected only between the IPAQ scores and performance on the task that tapped into the mental manipulation stage of visuospatial working memory processing.

Regarding the validity of the three tasks, all participants spent less time but made more accurate responses on the SST than on the VST and MRT, suggesting different cognitive demands of the three task. Storage processing as examined in the SST reflected the effort needed to simply recognize the image and therefore required lower cognitive demand than the general visuospatial working memory task, which required not only remembering the original image but also mentally rotating it (Guo et al., 2016). In addition, participants spent the most time on the MRT and made more errors on this task than on the SST. Compared with the SST and VST, the MRT required primarily online manipulation processing, which took participants longer. These results suggested that the short-term memory storage task was much easier than the mental rotation task for college students.

Consistent with previous studies, participation in PA was associated with better cognitive function (Colcombe & Kramer, 2003; Erickson & Kramer, 2009; Hillman, Erickson & Kramer, 2008; Smith et al., 2010). In the current study, the HG exhibited better visuospatial working memory in that their processing speed was faster than that for the LG. However, we observed no difference in response accuracy between groups, demonstrating no speed–accuracy tradeoff and indicating that speed underpinned the superior task performance of the more physically active group. These results suggest that participants who engage in PA take less time to ensure accurate performance of visuospatial working memory. A previously published meta-analysis partly supports our results, with the authors suggesting that acute exercise has a large beneficial effect on the response speed in a working memory task but a low to moderate detrimental effect on accuracy (McMorris et al., 2011). Therefore, we can conclude that the facilitated response speed was related to participation in PA.

A novel aspect of the present study was that we analyzed the contribution of the temporal phases of visuospatial working memory by using two tasks to distinguish the two stages of visuospatial working memory processing and explored their relationship to PA. Our results showed that the HG responded significantly faster than the LG on the online mental manipulation task (MRT), without response accuracy costs. However, we found no significant difference between groups for either RT or response accuracy in the short-term storage task (SST). Considering our results on the general visuospatial memory task (VST), these findings suggested that the relationship between PA and visuospatial working memory existed due to the enhancement in mental manipulation processing, although the results of the SST could not exclude a role for short-term storage in the relationship. This finding was inconsistent with our hypothesis and with previous research that found that older adults with higher aerobic fitness who walked regularly showed better performance on short-term memory tasks (Voss et al., 2013). One possibility for our lack of finding an effect on short-term memory storage may be related to test sensitivity. That is, the SST used here may be too simple for undergraduate students (compared with older adults) to show a significant relationship to PA. In addition, our finding may support the idea that PA selectively improves cognitive function requiring higher cognitive demands (Biddle & Asare, 2011; Tomporowski, Lambourne & Okumura, 2011). Such a selective improvement has been shown by many previous studies, some of which used the Flanker task to examine the relationship between PA and inhibitory control. Their results showed that participants who engage in PA respond faster and more accurately, especially for trials needing more cognitive control (Hillman et al., 2006; Kramer et al., 1999).

Another result that was inconsistent with our hypothesis was that we failed to find an effect of sex on visuospatial working memory. In the present study, the three tasks all required spatial perception processing, and we expected the males to outperform the females (De Lisi & Cammarano, 1996; Masters & Sanders, 1993). One plausible explanation for our lack of a sex effect may be that our study had fewer females than males. For example, in some previous studies reporting significant sex differences in visuospatial abilities, the numbers of males and females were approximately equal (Geiser, Lehmann & Eid, 2006; Kaufman, 2007). However, a neuroimaging study investigating sex differences in brain activity during a mental rotation task also found no significant difference in behavioral performance between the sexes (Hugdahl, Thomsen & Ersland, 2006). The authors proposed that a plausible reason for this outcome may be a lower sensitivity of the behavioral data compared with that of BOLD activation. Thus, future studies using neuroimaging techniques may provide important results regarding sex differences in visuospatial working memory.

Our results revealed a linear negative correlation between RT on the MRT and IPAQ scores, suggesting that participation in PA could improve the response speed of manipulation processing. However, we did not found a significant correlation between behavioral performances on the VST or SST and IPAQ scores. These findings provided further evidence for the selective improvement of PA on the manipulation stage of memory processing, which requires more cognitive demand than the short-term memory storage stage.

One limitation of the study concerns the difficulty of drawing a causal relationship based on the current experimental design. However, this preliminary exploration on the relationship between PA and visuospatial working memory indicates that further research using a longitudinal intervention design would help disentangle the direct effects of PA on visuospatial working memory. As previously mentioned, our finding of no significant relationship between PA and performance on the SST may be due to our use of a relatively simple task; more difficult tasks should be considered in future research. In addition, the classification of the participants was based on their answers to a questionnaire, a subjective measurement. Future studies should use objective measures to quantify the level of physical activity, such as the data provided by accelerometers or pedometers.

## Conclusions

In conclusion, increased PA was associated with better visuospatial working memory in college students. Furthermore, PA was selectively related to the manipulation stage of visuospatial working memory processing, which required high cognitive demand. The improved visuospatial working memory in the group of participants with higher PA was derived from a facilitation in manipulation processing speed without a loss of accuracy. These findings provide new insight into the relationship between PA and visuospatial working memory from the perspective of consecutive cognitive processing and suggest that PA may be significantly related to better visuospatial working memory in college students.

##  Supplemental Information

10.7717/peerj.3430/supp-1 Supplemental Information 1 Data Basic information and visuospatial working memory scores of participants

## Additional Information and Declarations

### Data Availability

The following information was supplied regarding data availability: The raw data has been supplied as a Supplementary File.
